# Narrative influence on support of a public policy: The case of nuclear power in The Netherlands

**DOI:** 10.1093/pnasnexus/pgae149

**Published:** 2024-04-09

**Authors:** Lotte de Lint, Maximilian Roßmann, Alexander Vostroknutov

**Affiliations:** Consumption and Healthy Lifestyles Group, Wageningen University and Research, 6706 KN Wageningen, The Netherlands; Department of History, Maastricht University, 6211 SZ Maastricht, The Netherlands; Department of Microeconomics and Public Economics, Maastricht University, 6211 LM Maastricht, The Netherlands

**Keywords:** narratives, cooperation, public goods, economic policy, technology ethics

## Abstract

We propose a new methodology to systematically transform presurveyed argument preferences into fictional narratives, that can help people to imagine the consequences of future events, and measure how they impact willingness to pay for a public policy. We apply narrative theory to construct two short narratives that depict an imaginary future, bleak due to climate change or energy dependence, and show experimentally that exposure to these narratives increases contributions in a Public Goods game, framed as payments toward the construction of new nuclear plant in The Netherlands. Our results suggest that fictional narratives can be used (and misused) as a tool of economic policy that allows conveying relevant information to people about complex issues. We discuss the ethical use of narratives and the value of their transparent construction for democratic will-formation and policy implementation when abstract factual information can be difficult to process or comprehend.

**Significance Statement**

We propose a methodology to construct fictional narratives that can help people to better understand the often complex arguments surrounding modern-day societal challenges and, consequently, make a better-informed choice. The method can be used to promote economic policies and to ensure that their implementation is not suffering from difficulties with understanding of their intended goals.

## Introduction

Complex societal issues such as global warming, pandemics, or demographic change pose the challenge for democracies to make and implement knowledge-based decisions. Simply providing more information to the population does not automatically enhance the depth of discourse or increase the acceptance of unpopular decisions. Similarly, the idea that people just lack information disguises the often-underlying value conflicts or ambiguous perspectives and can generally be considered outdated (1). People do not “assimilate, or experience science different from other elements of knowledge or judgment” (1) and facts only matter within the frames and contexts that structure a policy discourse. That is why large parts of political communication are mainly about setting frames that stage evidence, action, and authority in favor of preferred positions.

The call for new narratives to address the aforementioned challenges is omnipresent and at the same time difficult to tackle. To explain the meaning of narratives in public discourse, Barth and Heath (2) suggested the homology that sentences become meaningful within a narrative, just as words make sense within a sentence. Understanding a policy discourse as the third level of meaning, therefore, reveals how dominant narratives give relevance to uttered sentences suggesting different pathways for action. To this end, it has been shown that varying narratives can change what facts, data, values, and research proposals are considered relevant in the assessment of a future technology in mass-media discourse (3–5) and at the level of group interaction (6). Thus, the purposeful construction of narratives could help mitigate conflicts and help people take a different perspective.

Narratives are considered useful in situations when some limits on understanding involved arguments and/or on attention create constraints for engagement in argumentative communication. Studies under the “homo narrans” paradigm suggest that narrative understanding is more efficient and “natural” and that narratives constitute a sociocultural prerequisite for being part of a community (7–9). Instead of reasoning about the evidence or consistency of arguments for or against policy options, narratives “simulate” the experience of how such facts might unfold within a context of action (10, 11). Given that processing factual information often requires skills and background knowledge that only a small part of the population shares, the purposeful use of narratives can help to include people in political debates and give relevance to neglected positions. However, using narratives in democratic will-formation or policy implementation demands an ethical assessment for each case, as we argue in the The ethical use of narratives in democratic will-formation and policy implementation section.

The study of new narratives that can enhance policy communication has been difficult because of the ambiguous meaning of the term “narrative” within different disciplines and discourses. In some literatures, narrative refers to a structure inherent in political or life-course discourses across different sites, media, and occasions that explains emergent conflicts and coalitions or reorientation. At the same time, social science approaches, such as the narrative policy framework (5, 12) or the narrative discourse analysis (13), compare frames or topics by word cooccurrence and study narrative structures that represent factual actors as heroes, villains, or helpers whose actions and success or failure suggests a moral or policy preference.^a^ The study of narratives by literary scholars does not exclude these approaches but, in contrast, tends to examine linguistic features and the cultural perception of specific text sections of books or defined corpora with more qualitative detail due to a different research interest (for an overview, see Ref. (16)). In our eyes, these research traditions do not exclude but complement each other and provide valuable distinctions for our narrative design in the Narrative construction section.

In this study, we test experimentally whether narratives can significantly impact currently relevant economic decision-making and policy. Specifically, we propose a method to systematically transform the presurveyed relevance and preferences for technology features in a population into *fictional narratives* (in our case two: 362 and 288 words) that significantly increase their willingness to pay (WTP) by highlighting and downplaying focused aspects of the public discourse. Notice that our aim is to check how people’s economic choices change in response to a narrative, but we do not consider whether text characteristics influence the oral and medial dissemination that precedes the narrative exposition. To test whether our narratives were successful at convincing people, we compared the contributions in a Public Goods game (17) between subjects who were exposed to a narrative and a control group (18, 19). We found that both narratives significantly increased the contributions by 18%, thus validating our hypothesis and methodology.

To conduct the study in a realistic policy context, we constructed two narratives related to the currently active discussion of the future of nuclear energy in The Netherlands. The most recent evaluation of public support in The Netherlands found that only 45% of Dutch citizens would want more nuclear generated energy (20). Conversely, multiple independent research teams have determined that increasing the nuclear power capacity in The Netherlands would be a welfare-enhancing policy (21, 22). The growth of nuclear power capacity in The Netherlands is contingent on public approval, given that government subsidies and guarantees are fundamental in securing the necessary finances for new nuclear power plants (21). This setting therefore provides a good testing ground for our methodology. The consequences of planning more nuclear plants are complex and highly uncertain. Thus, it is reasonable to believe that some people might be overwhelmed or confused about this debate and that narratives can help them to make sense of some central issues at stake when people decide on the future of nuclear energy.^b^

## Methods

The experiment was approved through the agreement between BEELab (Maastricht University) and the Ethical Review Committee Inner City Faculties (Maastricht University). No number was issued given the agreement to approve standard economics experiments. Written consent was not directly obtained from participants since the participants were registered survey takers on Prolific.co and they gave consent to participate in surveys when they registered there.

The experiment was conducted in Dutch using the combination of a Qualtrics survey and subjects recruited at Prolific. Overall, there were 450 participants, of whom 405 were unique (45 subjects participated in two experimental sessions). Participants were recruited with one participation condition: Dutch as a first language. No pilot experiments were run or participants discarded. For demographic information across treatments see Section S10.

**Fig. 1.**
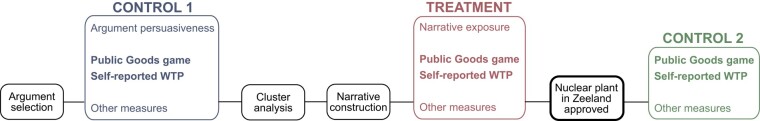
Timeline of the experiment from left to right and the point in time when the nuclear plant in Zeeland was approved by Dutch government.

Figure 1 shows the timeline of the experiment from left to right. Section S7 contains all instructions (for Dutch version see Section S8). In the first step, we selected arguments related to nuclear energy that were collected from the most popular mass media (23) with the idea to use the most persuasive arguments in the narrative construction. We analyzed articles on nuclear power published by major Dutch news broadcasters as well as those produced by Google searches on “Arguments against nuclear power” and “Arguments in favor of nuclear power.” We collected all proposed arguments from these articles, grouped them thematically to lower the number of arguments, and formulated core messages of each thematic group. Twelve final arguments resulted from this analysis (see survey questions in Section S7.1).

We used the 12 resulting arguments to evaluate their persuasiveness in Control 1, the first experiment with 150 subjects, who were given the Argument Persuasiveness questionnaire for that purpose (see Section S7.1). In Control 1, we also elicited subjects’ *contributions in the Public Goods game* (incentivized with real money and framed as paying taxes for building a new nuclear plant that leads to lower electricity prices for everyone; see Section S7.2) and unincentivized *self-reported willingness to pay for nuclear energy* (self-reported WTP or SWTP; see Section S7.3). We use both measures to test the effects of narrative exposure. Notice that subjects in Control 1 chose in the Public Goods game and reported their WTP without being exposed to any narratives. We use the choices in Control 1 as a benchmark to compare with subjects who were exposed to narratives in another treatment.

In the next step, we used the Argument Persuasiveness questionnaire from Control 1 to construct narratives. First, we conducted cluster analysis and found two clusters of subjects different in terms of the persuasiveness of the 12 arguments about nuclear power. Then, for each group we chose the most persuasive arguments (though, see Section S1.3 for details) and constructed two narratives that take into account the arguments chosen for each cluster.

In Treatment, we exposed 75 subjects to each narrative (150 subjects overall: 105 new subjects and 45 repeated from Control 1, see also the Additional checks section for more details) and then elicited their contributions in the Public Goods game and their self-reported WTP (along with other measures). These choices of participants in Treatment are our main variables of interest that we compare to the same choices in control treatments, where participants were not exposed to narratives. This comparison allows us to verify the effects of our constructed narratives on policy relevant behavior.

After Treatment, we also ran Control 2 (150 new subjects), which was the same as Control 1 but without the Argument Persuasiveness questionnaire. The goal of Control 2 was to make sure that any effect we observe between Control 1 and Treatment is not the result of some (unknown) trend in people’s opinions or change in their preferences that could have resulted from some event that took place between measurements. We, thus, aim to detect the effect of narrative exposure by comparing the contributions in the Public Goods game and SWTP between Treatment and the two controls.^c^

Finally, a major unexpected event related to nuclear energy in The Netherlands did in fact take place between Treatment and Control 2. The Dutch government—after a long public debate and years of planning—has approved the construction of nuclear power plants in Zeeland. This presented us with a perfect robustness check to test if this event had an effect on the contributions and views expressed by our subjects.

## Narrative construction

This section outlines our method to translate arguments with persuasive intent into narrative form. We understand a narrative as a representation of a sequence of events (story) held together by the plot (24). Engaging with a narrative means to imagine what is according to the text (explicitly and implicitly) is to be imagined (6, 25).^d^ A text thus *calibrates* what the audience imagines and allows a group to jointly explore, experience, and discuss fictional worlds from the inside instead of only assessing the language or truth value of claims.

**Fig. 2.**
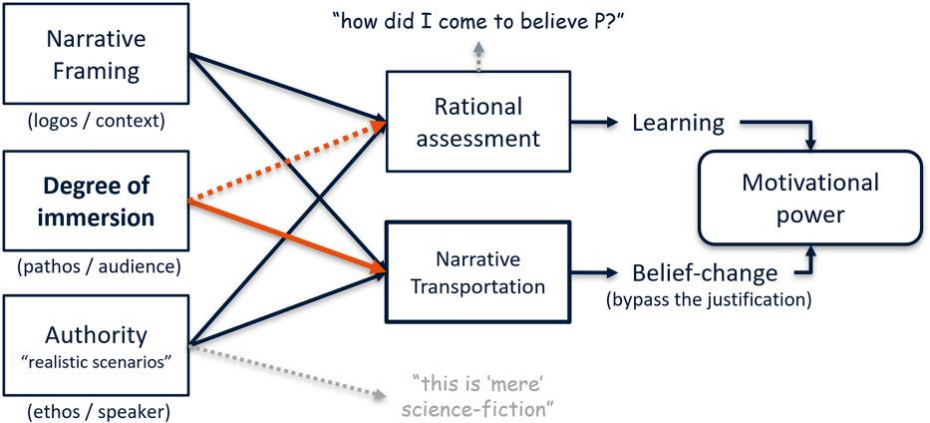
Three rhetorical dimensions of our study that guide deliberation or manipulation of narratives.

Crafting a (technology) narrative is an iterative process on the level of the story (i.e. how emphasized technology features become crucial for the fictional course of action) and discourse (what words, voices, and other means suit to best tell this story for the audience). The process is iterative as characters and the setting define the story world and potential actions or events in the plot as well as provide the voice, authority, and sympathy for the author to speak to an audience. Iteration is also important to refine what information to make explicit, e.g. about the protagonist or the setting, and what gaps are to be filled by the audience. The general implicit rule for engaging with narratives is to understand everything stated explicitly as being relevant for the overall plot and its consequent moral or policy implications. As outlined in Fig. 2, we aim to control three dimensions for persuasive narrative design on the basis of Aristotle’s rhetorical triangle, namely the plot and framing (logos), immersion (pathos), and authority (ethos).

Central to a persuasive narrative is deciding what the plot is about, or respectively what arguments or concepts to focus on. In contrast to a defense speech or scientific debate, the narrative carries the argument and moral implication by the logic of represented sequential events. One event follows the other and, within the limitations of a coherent plot, the narrators decides what to focus on and what to keep out of the frame. A popular way for plot construction is representing certain key events or actions as a deviation from a stated normalcy that calls for a resolution. To do so, the text engages the audience’s ideas about routines, social relationships, or relationships with technology, portrays them as what is normal, and incites imagining a deviation with meaningful consequences—e.g. someone foreign arrives or leaves town or the discovery of new technology leads to tensions in established routines and require further investigation. Closure then restores the old or suggests a new normalcy to resolve this emergent tension in the course of the story, but closure is no necessity. Still, in order to draft a narrative *about* nuclear power, features of nuclear power must not only be part of the setting or in a relationship to characters but they must be relevant for the plot.^e^

**Fig. 3.**
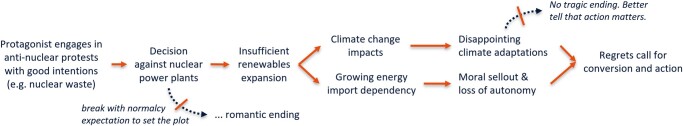
The two stories outline causal sequences of events. The stories end with a moral or policy implication but due to different arguments as represented in the plot.

Persuasion and behavioural change do not require explicit claims about reality to simulate experiences about real-world settings. Still, readers must employ beliefs to their imagination to draw inferences that affect long-term behavior (27). If values, such as political independence or corporate profits, do not matter to the reader, but are thought to influence attitudes to technology, the narrative must additionally depict this, e.g. by representing consequences of their ignorance being undesirable. For our case study, we transformed the two clustered argument preferences into causal sequences of events. We use flashbacks to the real “anti-nuclear movement,” the slow expansion of renewable energy in Europe, and The Netherlands’s beautiful landscape as “indices” (2) that “root” (28) the audience’s imagination (see Fig. 3). We follow (10, 12) in measuring the congruence of the depicted world and participants previous knowledge and attitudes in two congruence factors.

We interpret a story’s emotional appeal (pathos) as the archieved depth of immersion and compliance with the story and framing. The general idea of narrative transportation and immersion is to simulate experiences in a fictional world that change the appreciator’s understanding of the real world. Steering the degree of immersion first and foremost depends on the text style and plot quality (10). The conscious handling of information provided by the text as the plot develops (focalization) creates suspense and surprise as the driving force for the imaginative engagement anticipating the course of action. Because style is a complex concept, we mainly focus on catalysts, relatable characters, and “immersive resistance.” Catalysts are words and descriptions that do not carry the events as the plot-nuclei do but guide attention, stretch the time engaging with certain events, and catalyze the mood and feeling (2). Relatable characters meet the audience and discourse, allowing to take perspective when they fear, fume, or suffer. Finally, the text must avoid “immersive resistance” due to general situations or events that readers just want to avoid imagining, such as rape or torture. In the end, however, good style can encompass a variety of text features and, in our functional understanding, complements the plot for the coherent experience of a narrative world. In our stories, the insufficient expansion of renewable energy breaks with the established normalcy expectation of not wanting nuclear power plants, which allows to represent counter-arguments and implies consequences the protagonist has to face: climate change and dependence on energy imports. Stylistic features, such as catalysts are highlighted in the annex.

We interpret ethos—which in classical rhetoric means representing the speaker as a trustworthy, virtuous, or social community member—as the authority and credibility given to the story so that it is not perceived as “mere science-fiction.”^f^ To generate “fictions of authority” (29), we use the voice and domain-specific authority of relatable stereotype characters that explain how the world works from their first-hand experience. Our protagonists are a teacher who knows and cares about the decline of values and a gardener who knows and cares about environmental devastation. Alternatives to claim authority can be scientists, “white old men,” “Silicon Valley visionaries,” or side stories and flashbacks in which actors have demonstrated virtues and good intentions through their actions. Other sources of authority that indicate a knowledgeable narrator and activate beliefs about reality are references to official documents, scientific references, or folk histories about the success of earlier disruptive technologies. In addition, studies in advertisement showed that perceived manipulative intention—as within one-sided instead of two-sided augmentations to communicate honesty and deny manipulative intentions—hinder persuasion (30, 31). Despite this, truth as personal relevance and truth as stylistic and logical coherence within complex textual structures can be sufficient to claim authority for the narrative transportation pathways (11).

## Argument selection and clustering

To determine the arguments about nuclear energy that should be used in the story, we analyze Argument Persuasiveness questions asked in Control 1 (based on Ref. (32), but see also Ref. (33) for similar approach). We evaluate whether the statements related to 12 arguments in the discussion around nuclear energy in The Netherlands (see Section S7.1) increase subjects’ WTP, together with others, for it. An agreement to pay more, given an argument, signals that the subject considers the argument as positive and can be persuaded by it. Similarly, disagreement signals that the subject takes the argument as a negative and will not be persuaded.

**Fig. 4.**
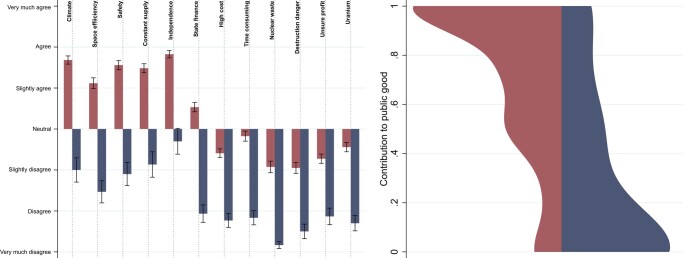
Left panel—Average answers to Argument Persuasiveness questions in the two clusters of subjects from Control 1. Error bars stand for SEs. See Section S7.1 for the descriptions of the questions. Right panel—Distributions of contributions to public good in the two clusters.

The left panel of Fig. 4 shows the average answers to Argument Persuasiveness questions in the two clusters of subjects in Control 1. The clusters were determined by the answers to the 12 questions and the analysis summarized in Section S2. Notice that the averages are strikingly different in the two clusters. While in the red cluster (120 subjects), subjects have positive view on the first six arguments and somewhat negative on the last six, in the blue cluster (30 subjects) the average answers are rather negative almost everywhere. This presents the case of a minority (20%) that sees nuclear energy as not a very reasonable solution, a minority that does not share the majority’s views that nuclear energy is a viable solution due to at least first six arguments. This disagreement on nuclear energy issues also manifests itself in the contributions to public good. The right panel of Fig. 4 shows the Gaussian-smoothed distributions of contributions in the two clusters. Most subjects in the majority red cluster contribute full amounts (average contribution 73%), whereas in the minority blue cluster most subjects contribute zero (average contribution 29%). The difference between the distributions is significant (rank-sum test: P<0.0001).

Given such a large disagreement between subjects in the two clusters, we decided to create two versions of the narrative structure designed to address the most persuasive arguments of each group. We chose argument Climate (“More nuclear power will help us to meet the climate goals”) for subjects in the red cluster and argument Independence (“More nuclear power plants increase our independence from other nations for our energy needs”) for subjects in the blue cluster as the most persuasive, main arguments. For each narrative, we chose three additional arguments out of which there was at least one positive and one negative. Section S7.3 details which phrases in the narratives correspond to which arguments. Notice that the choice of arguments for narratives does not have to follow the schema that we used. We chose the most persuasive arguments for our narratives to have the proof-of-the-concept, or to test if we can have an effect on public good contributions at all. However, in practice, researchers can follow other methods and choose arguments that need to be addressed for some other reasons not related to their persuasive power.

## Public good contributions

**Fig. 5.**
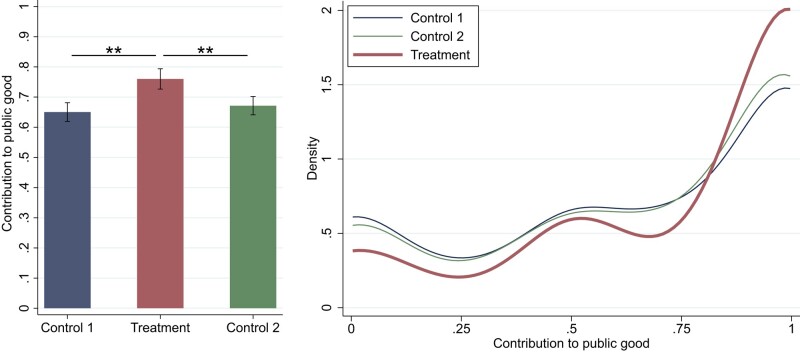
Left panel—Average percentages of the endowment contributed to public good by experimental session (**P<0.05). Right panel—Distributions of contributions to public good.

The main effect we study is the influence of narrative exposure on contributions to public good. Thus, in this section, we compare contributions across experimental sessions. First, we consider the effect of the general narrative structure on contributions. To test that, we pool together the data for the two versions of the narrative that have the same structure but are different in specific details. The left panel of Fig. 5 shows the average proportions of the endowments contributed (further *contributions to public good*) for all data in Control 1, Treatment, and Control 2. The averages are 0.65, 0.77, and 0.67, respectively. The average contributions in Treatment are 18% higher than in both controls, which is a significant increase (rank-sum test—Control 1: P=0.0103; Control 2: P=0.0243).

The right panel of Fig. 5 shows the Gaussian-smoothed distributions of contributions in the three experimental sessions. Notice that the distributions in both controls are almost identical, which suggests that this is a good measure that is stable in time (at least in the context of nuclear energy) even despite the fact that between the two control sessions a significant change in the debate over nuclear energy had occurred (see below). Next, notice that in Treatment more subjects than in controls choose to contribute 100% of the endowment and fewer subjects choose to contribute smaller amounts. This suggests that the general narrative structure inspires subjects, who would contribute less without reading the narrative, to contribute full amounts. Both the increase in the proportions of subjects who contribute full amounts and the decrease in the proportions of subjects who contribute less are significantly different between Treatment and controls (see Section S4 for the analysis).

Next, we consider separately the effects of the two versions of the narrative. The analysis presented in Section S3 shows that the average contributions for Narratives 1 and 2 are almost identical and are both significantly higher than in controls (in Control 1 at 5% level and at 10% level in Control 2 for both narratives). Similarly, the distributions of contributions for the two narratives are essentially the same. These observations suggest that the specific details of the narratives exert much less effect on contributions than the general narrative structure. Notice that from our data we cannot uncover the effects of the two narratives on the specific clusters, since we did not collect the survey answers in Treatment to divide participants into them. More research is needed to understand this influence.

## Effectiveness of narratives

Despite the seeming irrelevance of the narrative details for the increased contributions to the public good on the population level, we find that some specific characteristics of the two narratives nonetheless did have differential effects on contributions. Though, these effects are not visible in the average contributions that are the same for the two narratives. So, we suggest that interpretations of these measures are used with caution.

Following previous studies on “narrative persuasion” and “narratives as tools for influencing policy change” (10, 12), we used five measures described in more detail in Section S7.4 to estimate how subjects perceived the narratives to, in the end, influence their WTP. Table 1 presents regression analyses of the effects of these measures on contributions. Notice that Congruence2, or agreement with the statement “The story was about what I think is important in the nuclear power debate,” had a significant positive impact on contributions in Narrative 1. Congruence2 was only a marginally significant predictor of contributions to the public good for Narrative 2. Manipulation, or agreement with the statement “The person in the story comes across as manipulative,” had a significant negative impact on contributions in Narrative 2.

**Table 1.** Ordinary least squares robust regressions of contribution to public good on five measures of perception of a narrative.

	Narrative 1		Narrative 2	
Congruence2	0.074^**^	0.091^***^	0.083^*^	0.048
	(0.029)	(0.030)	(0.042)	(0.033)
Manipulation	− 0.009	− 0.016	− 0.090^**^	− 0.068^**^
	(0.032)	(0.033)	(0.042)	(0.033)
Congruence1	0.018		0.011	
	(0.020)		(0.022)	
Trust	− 0.033		− 0.039	
	(0.044)		(0.057)	
Identification	0.045		− 0.039	
	(0.028)		(0.035)	
Constant	0.356	0.374^**^	0.953^**^	0.728^***^
	(0.272)	(0.178)	(0.360)	(0.213)
*n* observations	56	56	49	49
$R^{2}$	0.25	0.19	0.17	0.14

*P<0.1; **P<0.05; ***P<0.01.

The first effect suggests that some subjects primarily liked Narrative 1 because it was about what they think is important and, as a result, contributed more to public good. The second effect suggests that some subjects found Narrative 2 manipulative and decreased their contributions.

To summarize, the analysis of measures like these can provide additional information about the effectiveness of the constructed narratives and can help to check if a narrative is too manipulative or congruent with the current perception of the issue.

## Self-reported willingness to pay

In some situations, it is not feasible to test narratives with an incentivized tasks like the Public Goods game, but only with simple questions. To check if our methodology works with such measures, we elicited self-reported WTP for nuclear energy on a 7-Likert scale (agree–disagree). The question we asked is the following: “I want to help pay for the construction of more nuclear power plants in the Netherlands.” The results for this measure reported in Section S5 are very similar to our analysis above: all main effects are the same. Though, the self-reported measures are more variable in time. This suggests nonetheless that simple questions can be used in practice to test the possible effect of narratives on economic behavior.

## Additional checks

In this section, we discuss some additional results and checks that can be important when applying our methodology. First, we notice that 45 subjects who took part in both Control 1 and Treatment did not react to narratives as much as other subjects who had not participated in Control 1 (repeated subjects did not change their average contribution to public good after the exposure to a narrative). Section S6 provides the analysis. We conjecture (without evidence) that this difference with repeated subjects is due to the fact that they have chosen in the Public Goods game twice and might have remembered what they chose before. Though, we still find that the same narrative characteristic (Congruence2) does influence the contributions to public good in the same way as for nonrepeated subjects.

Second, we would like to emphasize that the Public Goods game in Control 1 was different from those in Treatment and Control 2. Specifically, in Control 1 subjects had endowment of 2 Euro, while in the other sessions the endowment was 1 Euro. This was done for the purpose of testing whether the amount of endowment matters for framed Public Goods games as this one. We found that the endowment had no effect on the results as the distributions of contributions in Control 1 and Control 2 are virtually identical (see Fig. 4). This suggests that Public Goods games framed for specific topic are not sensitive to the size of the endowment (at least as long as it is not too large).

Third, we would like to mention that between Treatment and Control 2 a significant development in the nuclear energy debate in The Netherlands took place. The government agreed to build two new nuclear plants in Zeeland, thus resolving the debate in favor of supporters of nuclear energy. It is important to keep track of such events, as they might influence subjects’ attitudes and WTP. Interestingly, we find that contributions to public good did not change due to this event (see Fig. 4). However, as we document in Section S5, the self-reported WTP did change between the two controls. This suggests that events related to the narratives might change subjects’ attitudes, though not all of them. We leave it to future research to understand this in more detail.

Fourth, we collected a lot of additional demographic information about our subjects including various measures of economic behavior. We found no significant effects of any demographic variables or other measures on contributions to public good, except for a slight effect of the propensity to follow norms that was measured using the task of Kimbrough and Vostroknutov (34). We find that the measured propensity to follow norms positively correlates with the contributions to public good in both controls separately (Spearman’s rank correlation: Control 1−ρ=.15;P=.0656; Control 2−ρ=.17;P=0.0367) and together (ρ=.16;P=0.0063). The correlation is not significant in Treatment, which suggests that subjects with low propensity to follow norms increase their contributions in Treatment in comparison to controls. Overall, the significant correlation in controls suggests that the behavior of subjects is driven by norms to some extent. We did not find a correlation of propensity to follow norms with the self-reported WTP.

## The ethical use of narratives in democratic will-formation and policy implementation

We have shown that our methodology can be used to change people’s behavior related to an economically important subject. But a serious concern arises that narratives created by this method can be used for nefarious purposes and manipulation. Notice that technologies for manipulating people’s opinions and preferences using narratives already exist in abundance and are widely used. For example, emotionally loaded stories used in marketing and TV commercials are almost identical in style to ours: they describe the feelings of fictional protagonists that inspire observers to buy certain products. Thus, anyone who wishes to create a narrative for nefarious purposes does not have to go through the complex method described here and can simply use more traditional means.

To discuss the appropriate use of narratives in economic policy, we distinguish the process of *democratic will-formation* from the subsequent *policy implementation*. This distinction is closely linked to the distinction of using narratives for *comprehension* or *persuasion:*

“Do I want to facilitate potential controversy through greater understanding or reduce potential controversy through greater acceptance? Can I justify manipulating my audience?” (35, p. 610)

The will-formation calls for maximized mutual understanding of perspectives and arguments and requires generating attention to ongoing debates and decision points. Narrative comprehension can support democratic will-formation, for example, by making the relevance of technology for different lifeworlds better understood. But still, the selection and encouraged dissemination of popular narratives might exclude unpopular ones from the debate because medial space and peoples’ attention are limited. Besides, it remains an open question if coexisting narratives deepen or mediate value conflicts.

Reaching consensus or agreement with narrative persuasion stands against the ideal of deliberative democracy’s “unforced force of the better argument”—unless one understands narratives that aim at comprehension as a means for including marginalized groups in an otherwise elitist discourse (36). An ethical perspective on means and action (deontology) might generally discourage the use of narratives due to the tenuous influence on people’s deliberation and autonomy, especially when only selected arguments and value preferences are represented (35). However, from the perspective of consequences (consequentialism), one can justify the use of narratives in will-formation by stating that the value of raising public awareness or even advocating for a specific or a neglected perspective in debates, such as for example nuclear waste management, is greater than the independent will-formation (37). As both, a healthy environment and democratic standards are perquisites for free will-formation and higher level organization of economic and political actors, one can in principle justify the use of persuasive narratives in will-formation by the end of maintaining systemic perquisites of people telling stories and democracy. Still, this requires the democratic discussion of individual cases.

Narrative persuasion can also aid in implementing democratic decisions at lower costs. The greater good of a healthier or more sustainable behavior might justify using narratives with persuasive intention as a form of “governance,” especially when actors with commercial interests dominate the arena. To better understand and assess cases when actors have ulterior motives or can use knowledge as power, virtue ethicists might call for more narrative literacy. This includes ways of how narratives disguise or selectively highlight information (framing), claim realism and relevance in their indices and references (authority), and bypass or trigger rational reflection (transportation).

The aim of this project is not only to suggest and test narratives as a tool for economic policy but also to better understand the existing role of narratives in personal psychology, formation of beliefs, and decision-making. Especially in policy-related fields, a hermeneutic of popular stories provides insights into how people make sense of policy or technology issues (3). Thus, taking such stories seriously and discussing persuasive features can help to include people who would have been otherwise excluded from a discourse that only relies on rational argumentation. Our approach also suggests to not reduce narratives only to a medium that conveys information and values into a rational deliberation but also as a tool for encouraging people to tell and listen to each other’s stories as part of empowerment and community creation.

Our project envisions to transform the construction of narratives for specific welfare-enhancing purposes into a transparent sequence of steps that everyone can verify and discuss in an analytical framework. Indeed, all ingredients of the narratives that we created have a well-documented and well-defined reason that can be traced to the responses of subjects in Control 1 and the general purpose of the narrative being constructed. Thus, our method provides *accountability* for the created narratives that is crucial for using narratives as a tool of economic policy. If a public institution chooses to use a fictional narrative, it can provide a report in public access that documents how this narrative was created and what purposes it serves. This transparency allows for arguing against the view that *all narratives* are used for pursuing nefarious purposes. If people know that the narrative was created by a public institution for a specific welfare-enhancing purpose in a transparent way, their use might become more legitimate. Overall, the accountability and transparency proposed by our method can help to fight misinformation rather than promulgate it. Still, using narratives and pointing to authorship and transparency is not a silver bullet either to overcome the broader legitimization issues in a population that is suspicious of democratic institutions, the sciences, and public mass-media discourse.

## Limitations and future directions

Given the novel methodology of this study, it inherently faces certain limitations, thereby paving the way for future research opportunities. Since a between-subject design was deployed, the study focuses on aggregate population-level effects of narrative exposure. Repeated measurement of a subgroup of our sample did not generate sensible data, potentially due to participant recognition of the experiment upon reexposure. This leaves a gap in understanding which participant groups were most strongly influenced by the narratives. The regression analysis indicates that participants experiencing narratives with high congruence and low manipulation were more inclined to contribute, yet this analysis method cannot test for a causal relationship. Future research could employ a within-subject design to understand better the differential impacts of various narratives on individuals with diverse prior attitudes.

Additionally, our experiment prioritized internal validity using a convenience sample. Future studies should aim to replicate these findings in a nationally representative sample to enhance the generalizability of the results. This is especially critical for applying this methodology to craft and assess narratives for public institutions.

Our experiment deployed a short temporal gap between narrative exposure and behavioral measurement. This contrasts with real-world scenarios where narrative exposure and subsequent behavioral or voting actions are often more temporally separated. Therefore, investigating the long-term effects of narrative exposure remains a crucial area for future research.

We acknowledge the inherent demand effects in narrative experiments, as narratives invariably include implicit moral directives. While our findings suggest that these demand effects did not significantly impact the internal validity of our experiment, as indicated by the consistent behavior of repeat participants, further research is needed to compare the influences of experimenter-driven vs. institutional narrative demands on behavior.

Our approach explains persuasion and behavioral change with narrative-triggered inferences of participants’ beliefs. Since mental states are basically opaque to both participants and external observers, we understand surveyed beliefs as both, uttered descriptions (“de re”) of, e.g. nuclear power to be a solution to energy independence, and as a participant’s truthful commitment (“de dicto”) to making such claims premise for subsequent discourse, imaginative exercise, and action in the public good game (38). We do not evaluate if the commitment is empirically true in the sense of matching mental states (that are opaque) but, just as with regular conversations, if it meets the social conventions of a survey context that allow for such understanding. We, therefore, interpret the surveyed change of argument agreement after narrative exposition as a change of commitment in reaction to narrative induced inferences of colligated descriptive claims.

Finally, we suggest future research to examine the effect of presented narrative features by systematic variation, their impact on dissemination, the specific mechanisms of narrative persuasion, and the use of large language models (LLMs), such as LLaMA2 or GPT3, to aid the systematic construction of narratives as described.

## Conclusion

In this study, we test a novel methodology that allows to construct fictional narratives targeted at a specific topic and a specific population. We show that the narratives we have constructed do increase contributions in the Public Goods game, specifically framed for the purpose, and also increase self-reported WTP for nuclear energy in The Netherlands. We showcase several diagnostic tools that can be used to check how well a narrative was perceived and its various effects on behavior and beliefs.

## Supplementary Material

pgae149_Supplementary_Data

## Data Availability

The data underlying this article are available in OSF at https://osf.io/ptqsd/.

## Notes

a Both deductively exploiting Vladimir Propp’s morphology of the folk tale (14). For theoretical models of language and preferences within economics and psychology see also the review (15).

b It is worth noting that we, as academics, refrain from assessing the usefulness of new nuclear power plants and are primarily interested in the possible role of fictional narratives in a very technical and yet identity-political discourse.

c The fact that Control 1 contained Argument Persuasiveness questionnaire, while Control 2 did not, does not present a problem for our analysis since the choices in Control 1 and Control 2 are virtually indistinguishable (see discussion below).

d In terms of Gérard Genette’s narrative triangle (26), narratologists distinguish the guided imagination practice as “narrative situation” from the set of instructions as “narrative discourse,” and situate the fictional setting, imagined characters, and their actions or concerning events in the “story world.”

e Likewise, there is an implicit convention in appreciating narratives to expect that any explicit detail matters for the overall plot or discourse that the story is considered to be part of Ref. (2). If you would, for example, make the protagonist a safety officer in a nuclear power plant or an opponent of nuclear power, the audience is encouraged to assume that this position or related stereotypes make a difference in the course of the story.

f Understanding science fiction as unpolitical or irrelevant for technology development would be an understatement of the genre, as, e.g. studies in vision assessment show (4). While science fiction often plays in distant futures or universes, good plots and interesting protagonists resonate with the problems, desires, and attitudes of their audience. Indices to existing scientific claims and technology, as typical for the genre, then suggest engaging with regarding beliefs about reality as being relevant to these matters.

## Previous Presentation

These results were never previously presented.

## References

[1] Wynne B . 1991. Knowledges in context. Sci Technol Hum Val. 16(1):111–121.

[2] Barthes R, Heath S. 1977. Image, music, text: essays. 13. [dr.] ed. London: Fontana.

[3] Grunwald A . 2020. The objects of technology assessment. Hermeneutic extension of consequentialist reasoning. J Responsible Innov. 7(1):96–112.

[4] Schneider C, Roßmann M, Losch A, Grunwald A. 2023. Transformative vision assessment and 3-D printing futures: a new approach of technology assessment to address grand societal challenges. IEEE Trans Eng Manag. 70(3):1089–1098.

[5] Shanahan EA, Jones MD, McBeth MK, Lane RR. 2013. An angel on the wind: how heroic policy narratives shape policy realities: narrative policy framework. Policy Stud J. 41(3):453–483.

[6] Roßmann M . 2021. Vision as make-believe: how narratives and models represent sociotechnical futures. J Responsible Innov. 8(1):70–93.

[7] Fisher WR . 1984. Narration as a human communication paradigm: the case of public moral argument. Commun Monogr. 51(1):1–22.

[8] MacIntyre AC . 2007. After virtue: a study in moral theory. 3rd ed. Notre Dame (IN): University of Notre Dame Press.

[9] Somers MR . 1994. The narrative constitution of identity: a relational and network approach. Theory Soc. 23(5):605–649.

[10] Green MC . 2004. Transportation into narrative worlds: the role of prior knowledge and perceived realism. Discourse Process. 38(2):247–266.

[11] Oatley K . 1999. Why fiction may be twice as true as fact: fiction as cognitive and emotional simulation. Rev Gen Psychol. 3(2):101–117.

[12] Crow D, Jones M. 2018. Narratives as tools for influencing policy change. Policy Politics. 46(2):217–234.

[13] Viehöver W . 2001. Diskurse als narrationen. In: Keller R, Hirseland A, Schneider W, Viehöver W, editors. *Handbuch Sozialwissenschaftliche Diskursanalyse: band I: theorien und Methoden*. Opladen: Leske+Budrich. p. 177–206.

[14] Propp VI . 1968. Morphology of the folktale. Vol. 9. Austin (TX): University of Texas Press.

[15] Capraro V, Halpern JY, Perc M. 2024. From outcome-based to language-based preferences. J Econ Lit. 62:115–154.

[16] Scholes R, Phelan J, Kellogg R. 2006. Narrative theory, 1966–2006: a narrative. In: *The nature of narrative*. New York (NY): Oxford University Press. p. 283–336.

[17] Isaac RM, Walker JM, Williams AW. 1994. Group size and the voluntary provision of public goods: experimental evidence utilizing large groups. J Public Econ. 54(1):1–36.

[18] Hillenbrand A, Verrina E. 2022. The asymmetric effect of narratives on prosocial behavior. Games Econ Behav. 135:241–270.

[19] Osborn J, Wilson BJ, Sherwood BR. 2015. Conduct in narrativized trust games. South Econ J. 81(3):562–597.

[20] Ipsos . 2022. *Inzicht in de opinievorming van Nederlanders over Kernenergie*. Amsterdam: Ipsos.

[21] Veenstra A, Li X, Mulder M. 2022. Economic value of nuclear power in future energy systems: required subsidy in various scenarios regarding future renewable generation and electricity demand. Available at SSRN 4226375.

[22] E.J. van Druten, *et al*. 2022. HCSS Rethink Zero Witteveen+Bos, eRisk Group: scenariostudie kernenergie. Technical report, Ministerie van Economische Zaken en Klimaat.

[23] McCombs M, Valenzuela S. 2020. Setting the agenda: mass media and public opinion. Medford (MA): John Wiley & Sons.

[24] Abbot HP . 2007 July. Story, plot, and narration. In: Herman D, editor. *The Cambridge companion to narrative*. 1st ed. Cambridge: Cambridge University Press. p. 39–51.

[25] Walton KL . 1990. Mimesis as make-believe: on the foundations of the representational arts. Cambridge (MA): Harvard University Press.

[26] Genette G . 1980. Narrative discourse: an essay in method. Ithaca (NY): Cornell University Press.

[27] Schellenberg S . 2013. Belief and desire in imagination and immersion. J Philos. 110(9):497–517.

[28] Minkkinen M, Zimmer MP, Mäntymäki M. 2023. Co-shaping an ecosystem for responsible AI: five types of expectation work in response to a technological frame. Inf Syst Front. 25:103–121.

[29] Lanser SS . 1992. Fictions of authority: women writers and narrative voice. Ithaca (NY): Cornell University Press.

[30] Cohen J, Tal-Or N, Mazor-Tregerman M. 2015. The tempering effect of transportation: exploring the effects of transportation and identification during exposure to controversial two-sided narratives: the tempering effect of transportation. J Commun. 65(2):237–258.

[31] O’Keefe DJ . 1999. How to handle opposing arguments in persuasive messages: a meta-analytic review of the effects of one-sided and two-sided messages. Ann Int Commun Assoc. 22(1):209–249.

[32] Zhao X, Strasser A, Cappella JN, Lerman C, Fishbein M. 2011. A measure of perceived argument strength: reliability and validity. Commun Methods Meas. 5(1):48–75. 25568663 10.1080/19312458.2010.547822 PMC4283835

[33] Watts S, Stenner P. 2005. Doing Q methodology: theory, method and interpretation. Qual Res Psychol. 2(1):67–91.

[34] Kimbrough E, Vostroknutov A. 2018. A portable method of eliciting respect for social norms. Econ Lett. 168:147–150.

[35] Dahlstrom MF, Ho SS. 2012. Ethical considerations of using narrative to communicate science. Sci Commun. 34(5):592–617.

[36] Kohn M . 2000. Language, power, and persuasion: toward a critique of deliberative democracy. Constellations. 7(3):408–429.

[37] Smeddinck U, Roßmann M. 2022. Narrative als Regulierung?—Grundlagen, Ansätze, Verfassungsrecht. Deutsches Verwaltungsblatt. 137(3):137–145.

[38] Brandom R . 2000. Articulating reasons: an introduction to inferentialism. Cambridge (MA): Harvard University Press.

