# Supplementary material for: Narrative influence on support of a public policy: The case of nuclear power in The Netherlands
Source: PNAS Nexus. 2024 Apr 9;3(4):pgae149. doi: 10.1093/pnasnexus/pgae149 (PMC11032192; doi:10.1093/pnasnexus/pgae149)
Supplement: pgae149_Supplementary_Data [file pgae149_supplementary_data.zip › PNASNEXUS-PNASNEXUS-2023-01078RRR-s01.pdf]

Supplementary Material

Narrative influence on support of a public policy: the case of nuclear power in the Netherlands

by Lotte de Lint, Maximilian Roßmann, Alexander Vostroknutov

S1 Narratives

S1.1 Narrative 1

A message from the future

We have known for a very long time that in order to avert global warming, we must cut our emissions. I did not see nuclear power to be a viable solution because of pending issues with its waste management. I must admit that I have personally fought against it for decades. Without a doubt, our efforts have had some results and we have successfully installed a lot of solar panels and wind turbines. If only the growth of our renewable energy sources had been adequate to shut down fossil fuel plants while maintaining a steady supply.

Now, 30 years later, I occasionally remember these times and start seeing things differently. When my kids and I bike in the tulip fields outside of town, they aren't exploring the lovely meadows of my youth. Nearby, where once was a beautiful lake, they only discover some dead trees, arid fields, and dirty trickles. I had been attempting to grow veggies next to our bike shed for a few years. It may seem sentimental, I wanted to instill in my children a little sense of kinship with our land and the natural world, just like my parents did. However, soon I gave up on this endeavor when it became clear that either it didn't rain at all during these scorching summers or that massive rains ruined a year's worth of labor. Everything in the garden died.

Personally, I have always fared well, and we have built up a certain prosperity. Of course, someplace had to provide the power for our cars, homes, and cities. It is only years later that I realize the extent of climate change when I see what's left of our gorgeous landscape. Technologies were available. But it's possible that we missed out on a safe and cleaner solution.

I regret much having underestimated how polluting our energy system was as I look back on my life. When we had the option, I regret that I was so stubborn and did not also consider nuclear plants for a reliable and secure energy source. I am sending you a message from the future: Do all in your power to avert climate change.

S1.2 Narrative 2

A message from the future

It was like an uneasy awakening when political dependencies put our homes' and companies' energy security at stake. Since there was a chance of a nuclear accident and the still unresolved waste management, I must acknowledge that I have spent decades opposing and fighting nuclear power. Additionally, nuclear power seemed to be no longer a viable option due to the rising affordability of renewable energy sources. If only the growth of our renewable energy sources had been adequate.

Now, 30 years later, I occasionally remember these times and start seeing things differently. In my profession, working with young adults, it is important to hold up and teach a hopeful and democratic prospect. But to be honest, this is getting more and more difficult. Some claim it began when we were given the option of freezing in the winter or making dubious bargains to secure electricity supplies. Of course, nobody wanted to wear caps and gloves at home in one's apartment. However, becoming more reliant on problematic dependencies to meet our unsaturable demand for steady energy turned out even worse. The result is seen every day in the news: Along the way, we have lost our credibility selling out more and more of our values. Our western values that united us – are they just empty phrases to whitewash our Western lifestyle? We have become so dependent on imported gas, coal, and oil that our freedom is now torn between unpredictable weather and resource reliance.

I wish to maintain my optimism despite the difficulties, both for my pupils and for my friends and family. But it's possible that we overlooked the option for a reliable and independent solution, as technologies were available.

I greatly regret not realizing how delicate, and dependent our energy system was earlier in my life. When we had the option, I regret not also considering innovative nuclear plants as a way to reach our aspiration of independence. I am sending you a message from the future: Do not underestimate dependencies of a steady energy supply.

S1.3 Mapping of narrative structure and arguments

In this section, we describe the specific elements of the narratives that were constructed from the general narrative structure and the arguments chosen for each narrative. The narratives in S1.1 and S1.2 are color-coded to represent different elements of the design.

For the general narrative structure we chose the story of a middle-class Dutch individual in the future, who used to be a denier of benefits of nuclear energy, but 30 years later he regrets not having done more in the past. The parts of the narratives that reflect the setting and the character development along these general lines are coded in green. Note that the settings are deliberately chosen to feel familiar to Dutch people. The process of realization of past mistakes (not doing more about nuclear energy in the past) and consequent regret are expressed with emotional reactions of the protagonist.

The arguments chosen for the two narratives are coded in red (negative) and blue (positive). We chose to have both positive and negative arguments in each narrative because previous studies (e.g., O'Keefe, 1999, *Annals of the International Communication Association,* 22:1, 209-249) suggest that the mixture comes across as more persuasive than when only one-sided arguments are presented (which is seen as an attempt at manipulation). At least two positive and one opposing argument, with a minimum of four, were selected for inclusion in each narrative. This was done to ensure the balance across arguments. One positive argument was chosen as the central focus of each narrative.

For Narrative 1 that corresponds to the red cluster on Figure 4, we chose positive arguments Climate, Constant supply, and Safety, and negative argument Nuclear waste. The logic of choosing these arguments was the following. The three positive arguments are ranked as the most persuasive in terms of willingness to pay for nuclear energy (red cluster on the left panel of Figure 4) if we do not take into account Independence, which we use for the other narrative. The negative argument Nuclear waste was chosen as the one having the most negative effect on subjects' willingness to pay for nuclear energy to counterbalance the positive arguments.

For Narrative 2 corresponding to the blue cluster, we chose positive arguments Independence and Constant supply and negative arguments Destruction danger, Nuclear waste, and High cost. As before, Independence and Constant supply are the arguments that inspire the highest desire to pay for nuclear energy in the blue cluster (highest average values, see left panel of Figure 4). Similarly, Destruction danger and Nuclear waste

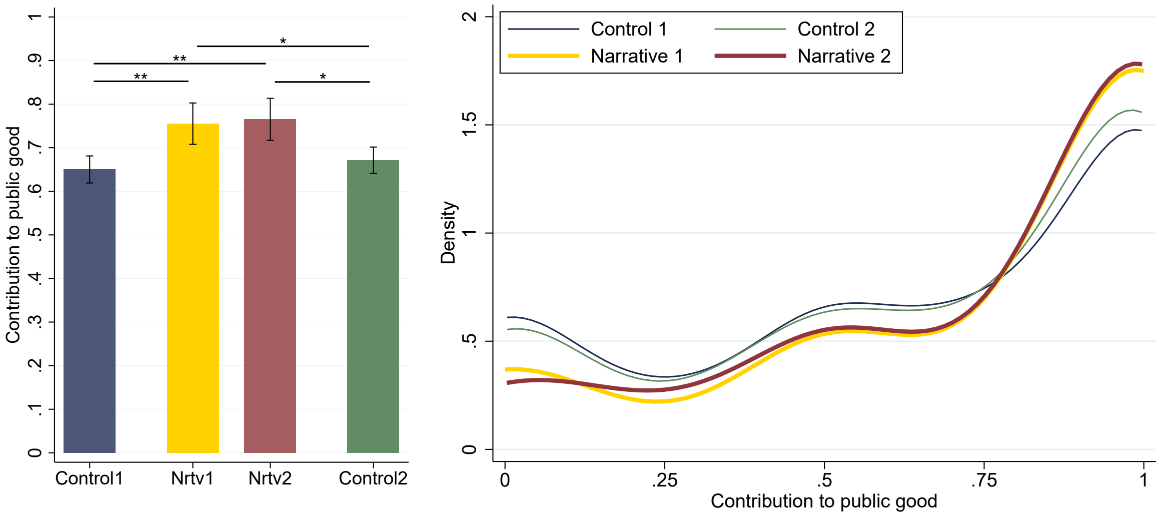

Fig. 6. Left Panel. Average percentages of the endowment contributed to public good in Control 1, Control 2, and Treatment divided into Narrative 1 and Narrative 2 ($*$ - $p < 0.1$; $**$ - $p < 0.05$). **Right Panel.** Distributions of contributions to public good.

are the arguments that people find the least persuasive in terms of paying for nuclear energy (roughly the least persuasive; we had to make some trade-offs).

S2 Details of cluster analysis

The optimal number of clusters was determined by employing the NbClust package in R, which uses a majority rule for 30 widely accepted tests (Charrad et al., 2014, *Journal of statistical software,* 61, 1-36). The optimal number of clusters for our dataset (Control 1) was two. Subsequently, the clusters were formed through K-means clustering.

S3 Analysis of the two narratives

The left panel of Figure 6 shows the contributions to public good in controls and separately for two Narratives presented in Treatment. The rank-sum tests between the contributions in Control 1 and the two narratives are significant at 5% ($p =$ .0489 and $p =$ .0377). The comparison of the contributions for each narrative with Control 2 are significant at 10% level ($p =$ .0715 and $p =$ .0808).

S4 Additional analysis of contributions

In the main text, we compared the distributions of contributions using non-parametric rank-sum tests that take into account whole distributions of variables. However, we also observed that the main shift in contributions in Treatment happened due to more subjects choosing full amounts to contribute. This suggests that we can run more detailed tests of the differences in contribution choices by looking at the proportions of subjects who choose certain amounts. For example, we can test if more subjects in Treatment chose full amount as compared to controls or some other proportion.

We define a dummy variable equal to 1 for subjects who made full contributions and 0 otherwise, and compare these variables across experimental sessions using binomial tests (we hypothesize that Treatment increases contributions). We find that the proportion of subjects who chose full amounts is significantly different between Control 1 and Treatment (one-sided $p =$ .0068; two-sided $p =$ .0135), between Control 2 and Treatment (one-sided $p =$ .0068; two-sided $p =$ .0135), and between both controls and Treatment (one-sided $p =$ .0028; two-sided $p =$ .0055). These differences are also significant if we consider two narratives separately. For comparisons of either Control 1 or 2 with either Narrative 1 or 2 we get one-sided $p < 0.0268$ and two-sided $p < 0.0537$ (see also the right panel of Figure 6). This suggests that both narratives drive significantly more subjects to choose full contribution than in controls.

We can also run similar tests for subjects who chose less than full amount. We pool both control sessions and compare the proportions of subjects who chose contributions less than or equal to 0%, 25%, 50%, and 75% between pooled controls and Treatment. We find that the proportions of subjects are significantly lower in Treatment than in controls. The p-values for the four comparisons are respectively $p =$ .0635, $p =$ .0424, $p =$ .0468, and $p =$ .0007 (one-sided binomial tests under the hypothesis that Treatment increases contributions; the two sided versions of the p-values are $p =$ .1270, $p =$ .0848, $p =$ .0949, and $p =$ .0014). Thus, we can conclude that our narrative structure has driven a significant proportion of subjects from contributing lower amounts to contributing full amount.

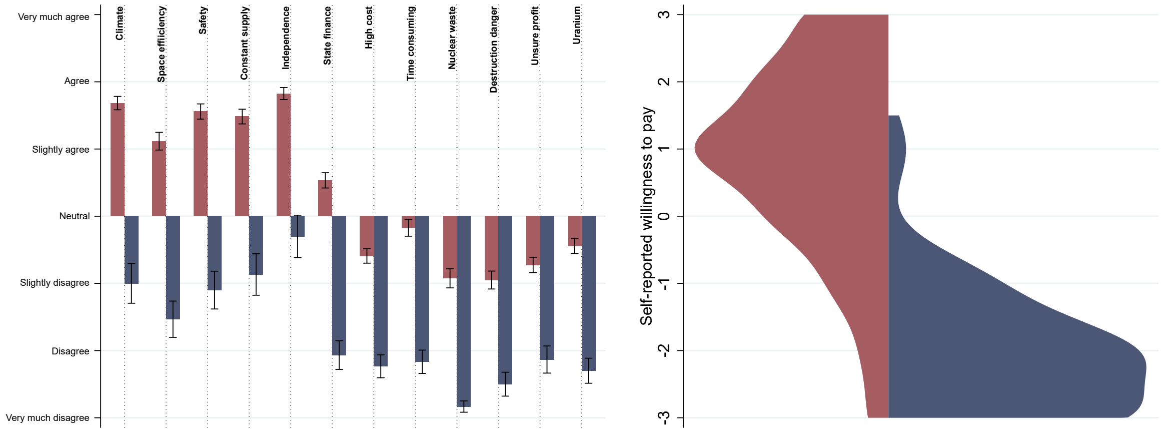

Fig. 7. Left Panel. Average answers to Argument Persuasiveness questions in the two clusters of subjects from Control 1. Error bars stand for standard errors. See Supplementary material S7.1 for the descriptions of the questions. **Right Panel.** Distributions of self-reported willingness to pay in the two clusters.

S5 Self-reported willingness to pay

Here, we present the same analysis as in the main text, only for the self-reported willingness to pay (SWTP) instead of contributions to public good. It is important to check whether we get similar results with this measure, as it is often the case that incentivized tasks (like the Public Goods game) are not possible to run.

Figure 7 shows the argument persuasiveness graph on the left (copied from the main text) and the distributions of SWTP in the two clusters. The distributions are significantly different (rank-sum test: $p < 0.0001$). The average SWTP in the red cluster is 0.84 and in the blue cluster −2.13. These results are in line with our findings for contributions to public good.

Next we focus on the comparisons of SWTP across experimental sessions. Figure 8 shows the same analysis as for the contributions. We see that SWTP in Control 1 is significantly different from Treatment (rank-sum test: $p = 0.0410$), and that no other comparisons are significant (the left panel of Figure 8). Notice that the two controls have rather different distributions of SWTP, and this is the reason why Treatment and Control 2 are not significantly different. We believe that the approval of new nuclear plants in Zeeland right before Control 2 might have to do with the change in Control 2 (though, we do not have evidence to support this claim).

The right panel of Figure 8 shows the distributions of SWTP in Control 1, 2, and Treatment. Here we observe an important difference from the analogous graph for contributions to public good. Notice that the narratives drive subjects to choose SWTP equal to 1 (slightly agree to pay), which is not the highest level possible as is the case with contributions. The distributions of SWTP in two controls look rather different, though not significantly different from each other.

When we compare the proportion of subjects who chose SWTP equal to 1 in different sessions, we find that one-sided binomial test (under the hypothesis that Treatment increases contributions) between pooled controls and Treatment is significant ($p = 0.0412$; two-sided $p = 0.0824$). This means that a significantly higher proportion of subjects chooses SWTP equal to 1 in Treatment as compared with controls. The comparison of proportions between Control 1 and Treatment yields $p = 0.0562$ and between Control 2 and Treatment we get $p = 0.0712$.

Also notice that the proportion of subjects who choose the lowest possible level of SWTP (−3) did not change in Treatment. This suggests that the narratives did not manage to change the personal opinions of the most vehement opponents of nuclear energy, but they did change their contributions to public good (we know from Figure 5 that zero contributions decrease in Treatment).

Notice as well that the narratives did have an effect on other subjects with negative SWTP. We see that much fewer subjects choose SWTP equal to −2 or −1 in Treatment as compared to Control 1. To see if this effect is significant, we compare the proportions of subjects who chose negative SWTP across sessions. One-sided binomial test between Control 1 and Treatment (under the hypothesis that Treatment increases contributions) gives a significant difference with $p = 0.0301$ (two-sided $p = 0.0602$). This shows that significantly less subjects chose negative SWTP in Treatment than in Control 1. The same test for Control 2 is not significant.

Next, we look at the two versions of the narrative structure separately. Figure 9 shows the results. On the right panel, we can observe that the two narratives create a slightly different distributions of SWTP. Narrative 1 seems to drive more subjects to choose SWTP equal to 1 than Narrative 2, though this difference is not significant. We find that the difference in distributions is only significant at 10% level between Control 1 and Narrative 1 (rank-sum test: $p = 0.0886$, see also the left panel of Figure 9).

When we look at the proportions of subjects choosing SWTP equal to 1 across narratives and controls, we find the only significant difference between Control 1 and Narrative 1 (one-sided binomial test under the hypothesis that Treatment increases contributions: $p = 0.0530$; two-sided $p = 0.1060$). This suggests that more subjects than in Control 1 choose SWTP equal to 1 after being treated with Narrative 1.

We find that the proportion of subjects who choose negative SWTP is significantly different between Control 1 and Narrative 1 (one-sided binomial test under the hypothesis that Treatment increases contributions: $p = 0.0336$; two-sided $p = 0.0672$). This shows that Narrative 1 significantly decreases the proportion of subjects with negative SWTP. Most likely, these subjects start choosing SWTP equal to 1 or higher.

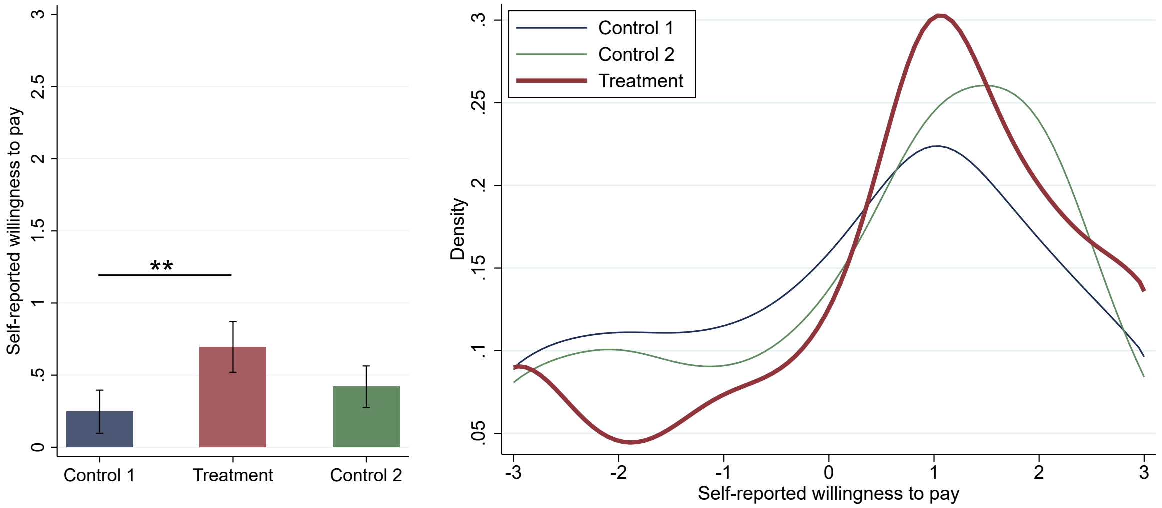

Fig. 8. Left Panel. Average self-reported willingness to pay by experimental session (** - $p < 0.05$). **Right Panel.** Distributions of self-reported willingness to pay.

Finally, we analyze the effects of narrative characteristics on SWTP. Table 2 presents the analysis in the same format as in the main text for the contributions to public good.

Table 2. OLS robust regressions of self-reported willingness to pay on five measures of perception of a narrative. * - $p < 0.1$; ** - $p < 0.05$; *** - $p < 0.01$.

	Narrative 1		Narrative 2	
Congruence2	.468***	.575***	.566**	.343*
	[.128]	[.134]	[.277]	[.187]
Manipulation	.005	-.081	-.401**	-.348*
	[.141]	[.145]	[.173]	[.183]
Congruence1	.151		-.150	
	[.117]		[.170]	
Trust	-.042		-.066	
	[.165]		[.265]	
Identification	.215		-.210	
	[.162]		[.267]	
Constant	-2.732**	-1.750**	.892	.029
	[1.262]	[.846]	[1.403]	[1.034]
N observations	56	56	49	49
R^2	.39	.31	.23	.17

We see that SWTP reacts to Narratives 1 and 2 in a way similar to contributions to public good. Specifically, in Narrative 1 subjects who find the narrative important (Congruence2), also choose higher SWTP. In Narrative 2, we observe the same negative effect of manipulation as with contributions. Thus, contributions to public good and SWTP are similarly affected by the details of each narrative.

S6 Repeated subjects

After we ran Control 1, we allowed subjects who took part in it to also participate in Treatment. The reason was to see the effect of being exposed to two experiments related to nuclear energy and whether participation in Control 1 had an effect on the behavior in Treatment.

Interestingly, we did find that 45 subjects who participated in both Control 1 and Treatment behaved differently. For example, these subjects did not significantly increase their contributions to public good in Treatment as compared to Control 1 (average contributions are .61 in Control 1 and 0.59 in Treatment). This may be so because subjects during Treatment remembered the amounts they contributed in Control 1. This is also suggested by the fact that we do find a significant effect of narrative characteristics on the contributions of repeated subjects. Specifically, the OLS robust regression of their contributions in Treatment on the five narrative characteristics (as in Table 1) gives a significant coefficient on Congruence2, similarly to our findings in the main text. This shows that some repeated subjects do respond to the congruence of the narratives and increase their contributions. However, such increase is not large enough to change the average contributions.

To assess whether self-selection bias influenced repeated participation in our experiment, we analyzed if nuclear power support indicators significantly predicted individuals' choosing to participate again. Table 3 presents these findings, revealing that only space efficiency and state finance arguments predict repeated participation at 10% level ($p < 0.1$). Consequently, this regression analysis provides no substantial evidence of systematic self-selection effects occurring in our study.

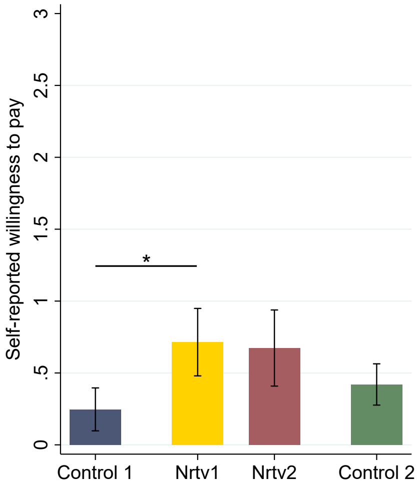

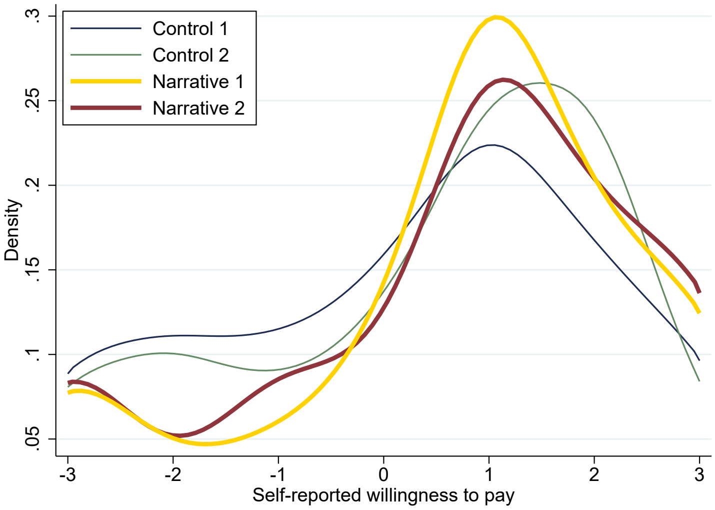

Fig. 9. Left Panel. Average self-reported willingness to pay in Control 1, Control 2, and Treatment divided into Narrative 1 and 2 ($*$ - $p < 0.1$). **Right Panel.** Distributions of self-reported willingness to pay.

Table 3. Binary logistic regression of repeated participation on nuclear power support indicators measured at Control1. * - $p < 0.1$; ** - $p < 0.05$; *** - $p < 0.01$.

WTP	-.339<br>[.746]
Self-reported WTP	.154<br>[.198]
Climate	-.013<br>[.188]
Space efficiency	0.332*<br>[.173]
Safety	-.283<br>[.201]
Constant supply	-.347<br>[.247]
Independence	.114<br>[.236]
State finance	.316*<br>[.186]
Cost	-.145<br>[.189]
Time consumption	-.006<br>[.174]
Nuclear waste	-.199<br>[.184]
Destruction	-.002<br>[.202]
Unsure profit	-.242<br>[.190]
Uranium	-.120<br>[.172]
Constant	-1.211**<br>[.600]
N observations	150
Pseudo R^2	.08

S7 Instructions (English translation)

S7.1 Argument persuasiveness

Please indicate how the statements below change your opinion about the following statement:

"I want to help pay for the construction of more nuclear power plants in the Netherlands."

(very much disagree; disagree; slightly disagree; neutral/no effect; slightly agree; agree; very much agree)

Climate More nuclear power will help us to meet the climate goals

Space efficiency Nuclear power plants are more space efficient than other green power sources like wind and solar

Safety Nuclear power is safer than energy from coal; It causes fewer deaths per unit of energy produced

Constant supply Unlike solar panels and wind turbines, nuclear power plants deliver a constant, reliable amount of energy.

Independence More nuclear power plants increase our independence from other nations for our energy needs.

State finance New nuclear power plants will have to be partially financed by the state because private parties see it as a high-risk investment.

High cost Building new nuclear power plants is expensive, with many recent plants going over budget.

Time consumption Nuclear power plants take a long time to build, and their benefits come late.

Nuclear waste Nuclear energy production creates radioactive waste

Destruction danger A nuclear power plant might fail and cause much damage

Unsure profit It is unsure whether a new nuclear power plant will be profitable since it is uncertain what the energy prices will be when the plant becomes operational.

Uranium Uranium reserves are limited.

S7.2 Public Goods game

The Dutch government is planning to build new nuclear power plants. To make this possible, we will have to help pay for its construction together.

In the hypothetical scenario of this study, you have 1 Euro to divide between the following choices. Option 1: You keep the money yourself or Option 2: You use the money to help pay for the construction of nuclear power plants.

The money put into Option 2 by all survey participants will be pooled. This money will become worth 1.5 times more, after which the amount increased in value will be divided equally among all participants.

This mechanism mimics the following future scenario:

- Investing in Option 2 creates more nuclear power plants;
- This results in a lower energy bill;
- Thereby, there is a money saving that is equal for everyone.

The participants in this study are random people from the Netherlands.

Your income = (Money from Option 1) + (Money from Option 2 from all participants * 1.5) / (Number of participants)

Calculation example:
All participants (and you) choose Option 1 ⇒ Your income: 1 Euro
All participants (and you) choose Option 2 ⇒ Your income: 1.5 Euro

So your income from Option 2 depends on how much the other research participants invest in nuclear power plants.

The outcome of this assignment will be paid to you in cash.

Make the same choice you would make in real life.

S7.3 Self-reported willingness to pay

What is your opinion about the following statement?

I want to help pay for the construction of more nuclear power plants in the Netherlands. (Very much agree; Agree; Slightly agree; Neutral; slightly disagree; Disagree; Very much disagree)

S7.4 Narrative characteristics

(1: not at all, ..., 7: very much)

Congruence 1 The story was consistent with how I view nuclear power.

Congruence 2 The story was about what I think is important in the nuclear power debate.

Trust The person in the story comes across as a trustworthy and honest person.

Manipulation The person in the story comes across as manipulative.

Identification I could identify with the person in the story.

S7.5 Rule-following task

In this question you can earn an additional small amount of money. You will decide how to allocate 10 balls between two buckets, a yellow bucket and a red bucket. For each ball you put in the red bucket, you will receive 1 cent, and for each ball you put in the yellow bucket, you will receive 2 cents.

The rule is to put the balls in the blue bucket.

Your payment will be based on your decisions: it is the sum of payments from the red and yellow buckets. You can choose any allocation of the balls. Your decision will have no consequences except for the payment as described above.

S8 Instructions (Dutch original)

S8.1 Argument persuasiveness

Geef aan hoe de hieronder genoemde statements uw mening veranderen over de volgende stelling:

“Ik wil meebetalen aan de bouw van meer kerncentrales in Nederland.”

Climate Meer kernenergie zal ons helpen de klimaatdoelstellingen te halen

Space efficiency Kerncentrales zijn ruimte-efficiënter dan andere duurzame energiebronnen zoals wind- en zonne-energie.

Safety Kernenergie is veiliger dan energie uit steenkool; het veroorzaakt minder doden per eenheid aan geproduceerde energie.

Constant supply Kerncentrales produceren een constantere, en daarmee een meer betrouwbare, energietoevoer dan zonnepanelen en windmolens.

Independence Meer kerncentrales zorgen ervoor dat we minder afhankelijk zijn van andere landen voor onze energiebehoefte.

State finance Nieuwe kerncentrales zullen deels door de staat gefinancierd moeten worden, omdat private partijen het als een risicovolle investering zien.

High cost De bouw van nieuwe kerncentrales kost veel geld, en veel recente centrales overschrijden het budget.

Time consumption De bouw van kerncentrales neemt veel tijd in beslag, waardoor we pas ver in de toekomst (over 5-10 jaar) profijt ervan hebben.

Nuclear waste De productie van kernenergie veroorzaakt radioactief afval.

Destruction danger Een kerncentrale kan problemen krijgen en veel schade aanrichten.

Unsure profit Het is niet zeker of een nieuwe kerncentrale rendabel zal zijn aangezien het onzeker is hoe hoog de energieprijzen zullen zijn op het moment dat de centrale operationeel wordt.

Uranium Uraniumreserves zijn beperkt en kunnen opraken.

S8.2 Public Goods game

Er wordt door de Nederlandse overheid plannen gemaakt om nieuwe kerncentrales te bouwen. Om dit mogelijk te maken zullen we samen moeten meebetalen aan de bouw hiervan.

In het hypothetische scenario van dit onderzoek heeft u 2 euro om te verdelen over de volgende keuzes. Optie 1: U houdt het geld zelf of Optie 2: U gebruikt het geld om mee te betalen aan de bouw van kerncentrales.

Het geld dat door alle onderzoeksdeelnemers in Optie 2 wordt gestopt zal worden samengevoegd. Dit geld wordt 1,5 keer meer waard, waarna het in waarde gestegen bedrag gelijk over alle deelnemers zal worden verdeeld.

Dit mechanisme bootst het volgende toekomstscenario na:

- Investeren in optie 2 zorgt voor meer kerncentrales.
- Dit veroorzaakt een lagere energierekening.
- Daarmee is er een geldbesparing die voor iedereen gelijk is.

De deelnemers uit dit onderzoek zijn willekeurige mensen uit Nederland.

Uw inkomsten = (Geld uit Optie 1) + (Geld uit Optie 2 van alle deelnemers * 1.5) / (Aantal deelnemers)

Rekenvoorbeeld:
Alle deelnemers (en u) kiezen Optie 1 ⇒ Jouw inkomsten: 2,-
Alle deelnemers (en u) kiezen Optie 2 ⇒ Jouw inkomsten: 3,-

Uw inkomsten uit optie 2 zijn dus afhankelijk van hoeveel de andere onderzoeksdeelnemers investeren in kerncentrales.

De uitkomst van deze opdracht wordt aan u uitbetaald in geld.

Maak de keuze die u in het echte leven ook zou maken.

S8.3 Self-reported willingness to pay

Wat is uw mening over de volgende stelling?

Ik wil meebetalen aan de bouw van meer kerncentrales in Nederland. (Zeer oneens; Oneens; Beetje oneens; Neutraal; Beetje eens; Eens; Zeer eens)

S8.4 Narrative characteristics

(1: helemal niet, ..., 7: heel erg)

Congruence 1 Het verhaal kwam overeen met hoe ik tegen kernenergie aankijk.

Congruence 2 Het verhaal ging over wat ik belangrijk vind in het kernenergiedebat.

Trust De persoon in het verhaal komt over als een betrouwbaar en eerlijk persoon.

Manipulation De persoon in het verhaal komt manipulatief over.

Identification Ik kon mij identificeren met de persoon uit het verhaal.

S8.5 Rule-following task

Met deze vraag kunt u een klein extra bedrag verdienen. U beslist hoe u 10 ballen verdeelt over twee emmers, een gele emmer en een rode emmer. Voor elke bal die u in de rode emmer doet, krijgt u 1 cent, en voor elke bal die u in de gele emmer doet, krijgt u 2 cent.

De regel is om de ballen in de rode emmer te doen.

Uw betaling wordt gebaseerd op uw beslissing: het is de som van het geld dat u krijgt uit de rode en de gele emmer. U kunt elke verdeling van de ballen kiezen. Uw beslissing zal geen gevolgen hebben behalve de betaling zoals hierboven beschreven.

S9 Narratives (Dutch original)

S9.1 Narrative 1

Een boodschap uit de toekomst

We weten al heel lang dat we onze uitstoot moeten verminderen om de opwarming van de aarde tegen te gaan. Vroeger zag ik kernenergie niet als een realistische oplossing vanwege het nucleaire afvalprobleem. Ik geef toe dat ik me er persoonlijk tientallen jaren tegen heb verzet. Met succes installeerden we echter wel velen zonnepanelen en windturbines. Onze acties tegen klimaatverandering hebben effect gehad, zonder twijfel. Was de energie uit onze hernieuwbare bronnen maar consistent en genoeg geweest om alle kolencentrales te kunnen sluiten.

Nu, 30 jaar later, denk ik af en toe terug aan vroeger en kijk ik anders tegen de dingen aan. Wanneer mijn kinderen en ik nu door de polders fietsen, verkennen we niet meer de mooie tulpenvelden uit mijn jeugd. Hier vlakbij, waar ooit een prachtige plas was, ontdekken ze nu alleen wat dode bomen, dorre velden en vieze stroompjes. Ik probeer nu al een paar jaar groenten te kweken naast ons fietsenhok. Het lijkt misschien sentimenteel, maar ik wilde mijn kinderen een beetje verbondenheid met ons land en de natuur bijbrengen, net zoals mijn ouders dat hebben gedaan. Maar ik heb het al snel opgegeven toen er duidelijk werd dat het ofwel helemaal niet regende tijdens de snikhete zomers ofwel dat massale regenval een jaar aan werk volledig wegspoelde. Alles in de tuin ging dood.

Persoonlijk heb ik altijd goed geboerd, en hebben we een zekere welvaart op kunnen bouwen. Natuurlijk moest de energie voor onze auto's, huizen en steden ergens vandaan komen. Als ik zie wat er nu nog maar over is van onze ooit zo prachtige natuur, besef ik me jaren later de schade die klimaatverandering heeft aangericht. Maar het is goed mogelijk dat we de optie voor een betrouwbare en onafhankelijke oplossing zijn mis gelopen, terwijl de technologie wel beschikbaar was.

Als ik terugkijk op mijn leven zie ik dat ons energiesysteem veel vervuilender was dan ik eerst dacht. Ik heb er spijt van dat ik te koppig was. Toen we de keus hadden, wilde ik gewoon niet inzien dat kernenergie ook een betrouwbare en veilige energiebron was. Daarom stuur ik je een boodschap uit de toekomst: Doe er alles aan om klimaatverandering te voorkomen.

S9.2 Narrative 2

Een boodschap uit de toekomst

Het was een shock toen ik me realiseerde dat de energiezekerheid van onze huizen en bedrijven opeens op het spel stond door politieke afhankelijkheden. Door de kans op een kernramp en het onopgeloste probleem van kernafval, moet ik toegeven dat ik me tientallen jaren verzet heb tegen kernenergie. Bovendien leek kernenergie niet langer een haalbare optie doordat duurzame energie steeds goedkoper werd. Was de groei van duurzame energie maar genoeg geweest.

Nu, 30 jaar later, denk ik af en toe terug aan vroeger en kijk ik anders tegen de dingen aan. Binnen mijn werk met jongvolwassenen, is het belangrijk om een hoopvol en democratisch perspectief te schetsen. Maar eerlijk gezegd wordt dit steeds moeilijker. Sommigen beweren dat het allemaal begon toen we de keuze kregen tussen kou leiden in de winter of dubieuze deals sluiten om onze toegang tot energie zeker te stellen. Natuurlijk zat niemand erop te wachten om thuis mutsen en wanten te moeten dragen. Echter, door onze onverzadigbare vraag naar een constante energietoevoer werden we steeds afhankelijker van problematische partijen. Het resultaat is dagelijks in het nieuws te zien: Gaandeweg hebben we onze geloofwaardigheid verloren door steeds meer van onze waarden aan de kant te schuiven. Onze westerse waarden die ons verenigden - zijn dat slechts holle uitdrukkingen om onze Westerse levensstijl goed te praten? We zijn zo afhankelijk geworden van geïmporteerd gas, steenkool en olie dat onze vrijheid nu wordt ingeperkt door onvoorspelbaar weer en onze afhankelijkheid van grondstoffen.

Ik wil ondanks alle problemen vasthouden aan mijn optimisme voor zowel voor mijn leerlingen als voor mijn vrienden en familie. Maar het is goed mogelijk dat we de optie voor een betrouwbare en onafhankelijke oplossing zijn mis gelopen, terwijl de technologie wel beschikbaar was.

Als ik terugkijk op mijn leven zie ik dat ons energiesysteem veel kwetsbaarder en afhankelijk was dan ik eerst dacht. Ik heb er spijt van dat ik te koppig was. Toen we de keus hadden, wilde ik gewoon niet inzien dat innovatieve kerncentrales een manier zijn om onafhankelijker te worden. Daarom stuur ik je een boodschap uit de toekomst: Onderschat niet hoe afhankelijk wij zijn voor onze stabiele energievoorziening.

S10 Demographics

Table 4. Demographic Information Per Experiment

**Variable**	**Value**	**Control 1**	**Treatment**	**Control 2**
Sex	Male	50%	50%	50%
	Female	50%	50%	50%
Age	Mean (SD)	31.0 (10.2)	30.2 (10.7)	28.1 (7.57)
Education	Doctoraat, PhD	2.0%	0.7%	2.0%
	Master (HBO / WO)	21%	21%	27%
	Bachelor (HBO / WO)	45%	47%	41%
	HAVO, VWO, MBO2-4	28%	27%	29%
	VMBO, HAVO / VWO onderbouw, MBO1	2.0%	3.0%	2.0%
	Primary school	0%	0.7%	0%
	Other	1.3%	0%	0%
Religiosity	True	29%	16%	22%
	False	71%	84%	78%
Ethnicity	North- and/or West-European (including North-America/ Oceania)	80%	81%	77%
	Other	20%	19%	23%
Political affiliation	BBB	0%	0%	0.7%
	CDA	1.3%	0%	0.7%
	ChristenUnie	1.3%	2.0%	1.3%
	D66	10%	10%	12%
	Forum voor Democratie	2.0%	0.7%	1.3%
	GroenLinks	18%	15%	16%
	Ja21	2.0%	4.0%	4.7%
	Partij voor de Dieren	11%	10%	6.0%
	PvdA	6.0%	5.3%	5.3%
	PVV	6.0%	4.0%	2.0%
	SGP	0.6%	1.3%	0.7%
	SP	4.0%	2.7%	3.3%
	Volt	6.0%	9.3%	11%
	VVD	5.3%	9.3%	8.0%
	Other	7.3%	4.0%	4.0%
	I do not know	19%	20%	23%
**Number of subjects**		150	150	150
